# Evaluation of deep vein thrombosis prophylaxis in a general hospital

**DOI:** 10.1590/1677-5449.007017

**Published:** 2018

**Authors:** Fátima Cristiane Lopes Goularte Farhat, Hellen Caroliny Torres Gregório, Rafaela Durrer Parolina de Carvalho

**Affiliations:** 1 Universidade Metodista de Piracicaba – UNIMEP, Faculdade de Ciências da Saúde, Curso de Farmácia, Piracicaba, SP, Brasil.; 2 Hospital Irmandade da Santa Casa de Misericórdia de Piracicaba, Serviço de Farmácia, Piracicaba, SP, Brasil.; 3 Universidade Estadual de Campinas – UNICAMP, Faculdade de Odontologia de Piracicaba – FOP, Piracicaba, SP, Brasil.

**Keywords:** venous thromboembolism, chemoprevention, heparin, hospital

## Abstract

**Background:**

Venous thromboembolism (VTE) is a cause for growing concern in hospitals, has great impact on morbidity and mortality in clinical and surgical patients, and is the leading cause of preventable hospital deaths. Although there are risk assessment models for hospital inpatients, prophylaxis is still underused or is administered incorrectly.

**Objectives:**

To assess the risk profile for VTE in recently hospitalized clinical and surgical patients and evaluate the thromboprophylactic measures implemented in the first 24 hours of hospitalization.

**Methods:**

Cross-sectional study conducted in a large general hospital in the state of São Paulo, Brazil, between March and July 2015. Padua and Caprini scores were used for risk stratification of clinical and surgical patients, respectively, while thromboprophylactic measures were analyzed for compliance with the recommendations contained in the 8th and 9th Consensus of the American College of Chest Physicians.

**Results:**

A total of 592 patients (62% clinical and 38% surgical) were assessed. Risk stratification revealed a need for chemoprophylaxis in 42% of clinical patients and 81% of surgical patients (51% high risk and 30% moderate risk). However, 54% of high-risk clinical patients, 85% of high-risk surgical patients, and 4% of moderate-risk surgical patients, who were free from contraindications, were actually given the correct prophylaxis in the first 24 hours of hospitalization.

**Conclusions:**

There is a need to improve patient safety in relation to VTE in the first hours of hospitalization, since there is underutilization of chemoprophylaxis, especially in high-risk clinical patients and moderate-risk surgical patients.

## INTRODUCTION

 Deep venous thrombosis (DVT) is the result of formation of thrombi in deep veins. It is most common in the lower limbs, but can involve the vena cava, the internal jugular veins, and upper limb veins. Thrombi may cause partial or total occlusion of the deep vein system, and the most serious immediate complication is pulmonary embolism (PE), which occurs after a thrombus detaches and obstructs blood flow in the pulmonary artery, with consequent cardiorespiratory events. 1 ^,^ 2

 Venous thromboembolism (VTE) comprises both of these related diseases, DVT and PE. Asymptomatic or clinically evident episodes in hospitalized patients can be associated with mortality. As such, VTE is considered the greatest cause of avoidable death in hospital settings. 1 ^,^ 3 ^-^ 5 It is a common disease among hospitalized patients, and can emerge as a complication of other clinical or surgical conditions, but it can also occur spontaneously in apparently healthy people. 6 According to the American College of Chest Physicians’ (ACCP) 8th consensus on VTE prevention, almost all hospitalized patients have at least one risk factor for VTE, and around 40% have three or more. Thromboprophylaxis is the initial strategy for improving the safety of hospitalized patients. 5

 Studies have confirmed that thromboprophylaxis is safe and effective. Measures such as early mobilization, graduated elastic compression stockings, intermittent pneumatic compression, and anticoagulants should be adopted rationally after appropriate risk stratification of patients, to avoid exposing them to unnecessary measures. It is also important not to omit such measures in patients for who they are indicated. 4 ^-^ 9

 However, it has been observed that thromboprophylaxis prescription rates are low and, when thromboprophylaxis is administered, it tends to be incorrect, despite the fact that protocols are available to guide health professionals. 6 ^,^ 10 ^-^ 12

 This study was conducted to evaluate the VTE risk profiles of recently-admitted clinical and surgical patients and to assess the thromboprophylactic measures administered during the first 24 hours after admission. 

## METHODS

 This cross-sectional, descriptive study was conducted at a large general hospital in upstate São Paulo, Brazil. Clinical and surgical patients over the age of 18 who remained in the institution for more than 24 hours were analyzed during the first 24 hours after admission from March to July of 2015. Pediatric patients, expectant women, and recently-delivered mothers, patients already being treated for thrombotic episodes, and patients for whom information was unavailable after three consecutive assessment attempts on at least 2 different days were all excluded. Patients admitted as surgical patients, but who did not undergo a surgical procedure within 48 hours of admission were reclassified and assessed as clinical patients. 

**Figure 1:**
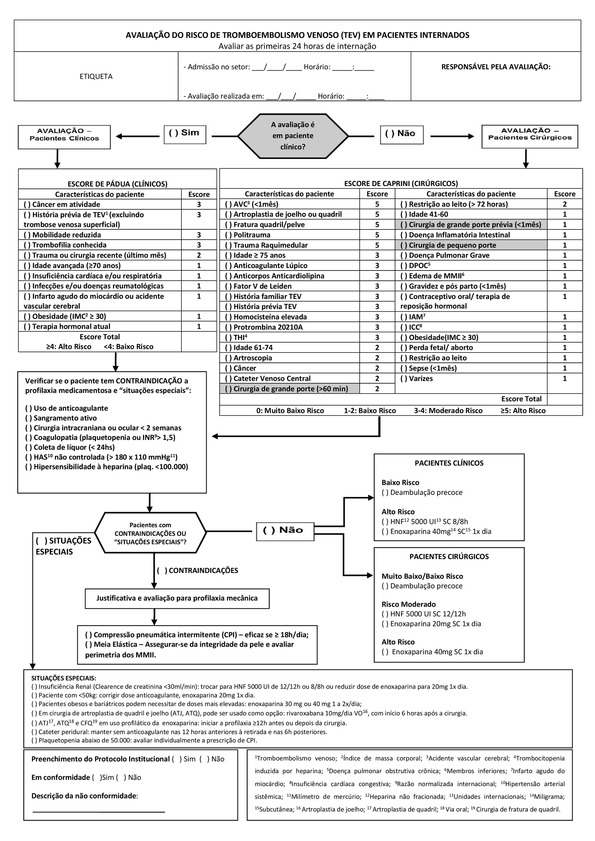
Flow chart (in Brazilian Portuguese) employed for VTE risk stratification and assessment of thromboprophylaxis administered to inpatients.

 A flow diagram ( Figure 1 ) for risk stratification and assessment of thromboprophylaxis was developed based on the ACCP recommendations for VTE prevention. 5 ^,^ 13 ^,^ 14 The Padua score 15 was adopted for risk stratification of clinical patients and the Caprini score 16 was used for surgical patients, while possible contraindications and conduct in special situations such as with obese patients (body mass index, BMI ≥ 30) and those with renal failure were also taken into account. Data on risk factors for VTE present during the first 24 hours after patients’ admission, thromboprophylactic measures adopted, contraindications against chemoprophylaxis, and special situations were collected from the healthcare team and the patients’ medical records. 

Table 1 lists the relationships between the scores adopted and risk stratification of clinical and surgical patients and also the thromboprophylaxis recommended by the ACCP in each case. Very low and low-risk surgical patients were classified as a single category since they have the same prophylaxis recommendation. 

**Table 1:** Risk stratification according to Padua and Caprini scores and thromboprophylaxis measures recommended by the American College of Chest Physicians.

**Risk stratification**		**Thromboprophylaxis**
		Padua score – Clinical patients
< 4 points	Low risk	Early mobilization
≥ 4 points	High risk	UFH: 5,000 UI 8/8h LMWH: 40 mg 1x/day Mechanical prophylaxis when chemoprophylaxis is contraindicated, reassess when bleeding risk reduces
		Caprini score – Surgical patients
0 points	Very low risk	Early mobilization
1-2 points	Low risk	Early mobilization
3-4 points	Moderate risk	UFH: 5,000 UI 12/12h LMWH: 20 mg 1x/day Mechanical prophylaxis when chemoprophylaxis is contraindicated, reassess when bleeding risk reduces
≥ 5 points	High risk	LMWH: 40 mg 1x/day Mechanical prophylaxis when chemoprophylaxis is contraindicated, reassess when bleeding risk reduces

LMWH, low molecular weight heparin; UFH, unfractionated heparin.

 After risk stratification of each patient, compliance of thromboprophylaxis with recommendations was assessed according to two criteria: prescription and daily dose of chemoprophylaxis. As such, conduct was considered compliant if two conditions were met, 1: if chemoprophylaxis was prescribed for cases in which it was necessary and 2: if the daily dose prescribed was correct for those patients for whom it was necessary, or it was not prescribed in cases in which it was unnecessary. Non-compliant conduct was defined as prescription of chemoprophylaxis for cases in which it was unnecessary or failure to prescribe or prescription of an incorrect dose for cases in which it was necessary. 

 The results were tabulated into 2x2 contingency tables and analyzed using the statistical package BioEstat 5.3, with the chi-square test. The significance level adopted was p < 0.05. 

 This study was approved by the Research Ethics Committee of Universidade Metodista de Piracicaba/UNIMEP (protocol number 36/2014) and was carried out at Hospital dos Fornecedores de Cana de Piracicaba (HFCP). 

## RESULTS

 A total of 592 patients were analyzed within 24 hours of admission: 369 (62%) clinical patients and 223 (38%) surgical patients. The prevalence of VTE risk factors and their levels of importance during the first few hours after admission of clinical and surgical patients are shown in Tables 2 and 3 , respectively. It will be observed that the 369 clinical patients had a total of 594 risk factors (mean of 1.6/patient), while the 223 surgical patients had a total of 575 (mean of 2.6/patient). 

**Table 2:** Risk factors identified in clinical patients, according to the Padua score.

**Risk factors**	**Score**	**n**	**% of patients**
Reduced mobility	3	214	58
Advanced age (≥ 70 years)	1	150	41
Infections and/or rheumatic diseases	1	87	24
Heart failure and/or respiratory	1	57	15
Obesity (BMI ≥ 30)	1	38	10
Active cancer	3	25	7
Acute myocardial infarction or stroke	1	12	3
Recent trauma or surgery (preceding month)	2	8	2
Current hormone therapy	1	2	0.5
Prior history of VTE (except superficial venous thrombosis)	3	1	0.3
Known thrombophilia	3	0	0
Total		594	

BMI, body mass index; VTE, venous thromboembolism.

**Table 3:** Risk factors identified in surgical patients, according to the Caprini score.

**Risk factors**	**Score**	**n**	**% of patients**
Major surgery (> 60 min)	2	134	60
Minor surgery	1	89	40
Age 41-60	1	87	39
Obesity (BMI ≥ 30)	1	73	33
Confined to bed	1	55	25
Age 61-74	2	41	18
Confined to bed (> 72 hours)	2	34	15
Age ≥ 75 years	3	22	10
Cancer	2	9	4
Central venous catheter	2	9	4
Lower limb edema	1	5	2
Knee or hip joint replacement	5	4	2
Arthroscopy	2	2	1
Polytrauma	5	2	1
AMI	1	2	1
Varicose veins	1	2	1
Family history of VTE	3	1	0.4
Prior major surgery (< 1 month)	1	1	0.4
COPD	1	1	0.4
Sepsis (< 1 month)	1	1	0.4
Oral contraceptive/hormone replacement therapy	1	1	0.4
Total		575	

COPD, chronic obstructive pulmonary disease; AMI, acute myocardial infarction; BMI, body mass index; VTE, venous thromboembolism.

Table 4 shows the profiles of the patients analyzed in terms of their risk stratification for VTE, mean number of risk factors/patient, the most prevalent risk factors in each group, and thromboprophylaxis recommendations according to the ACCP. 5 ^,^ 13 ^,^ 14 It can be observed that as risk of VTE rises, the mean number of associated risk factors per patient also rises, and the prevalence of factors with higher scores on the risk scales also rises. 

**Table 4:** Profiles of clinical and surgical patients after risk stratification for venous thromboembolism, mean number of risk factors per patient, most prevalent risk factors, and prophylaxis recommended by the American College of Chest Physicians.

**Risk stratification**	**n (%)**	**Mean number of risk factors per patient (range)**	**Most prevalent risk factors** **in patients analyzed**	**Thromboprophylaxis recommended**
Clinical patients	369	1.6		
Low risk	215 (58%)	0.9 (0-3)	Age > 70	Early mobilization
High risk	154 (42%)	2.6 (2-4)	Reduced mobility, age > 70, and active infections or rheumatic diseases	UFH: 5,000 UI 8/8h LMWH: 40 mg 1x/day
Surgical patients	223	2.6		
Very low risk/low risk	42 (19%)	1.6 (1-2)	Minor surgery, age 41-60	Early mobilization
Moderate risk	68 (30%)	2.4 (1-3)	Minor or major surgery, age 41-60, confined to bed	UFH: 5,000 UI 12/12h LMWH: 20 mg 1x/day
High risk	113 (51%)	3.1 (2-5)	Major surgery, obesity, age 41-60 years	LMWH: 40 mg 1x/day

LMWH, low molecular weight heparin; UFH, unfractionated heparin.

 The stratification process identified 154 (42%) clinical patients as at high risk, and 68 (30%) and 113 (51%) surgical patients as at moderate and high risk of VTE, respectively. It can also be observed that chemoprophylaxis was indicated for 335 (57%) patients in the whole sample in the first 24 hours after admission; 42% (154) of the clinical patients and 81% (181) of the surgical patients. 

 In contrast, there was evidence of contraindications to chemoprophylaxis in just 18 (3%) patients: 14 clinical patients (3.8%) and four surgical patients (1.8%). The reasons for contraindication identified are shown in Table 5 . In all of these cases, it was observed that lower limb motor physiotherapy was prescribed 2 to 3 times per day, probably as a thromboprophylactic measure. 

**Table 5:** Contraindications against chemoprophylaxis in patients analyzed.

**Contraindication**	**n**	**%**
On anticoagulants	11	61
Active bleeding	4	22
Uncontrolled SAH (> 180 x 110 mmHg)	2	11
Thrombocytopenia	1	6
Total	18	100

SAH, systemic arterial hypertension.

Table 6 lists the compliance of the thromboprophylaxis measures adopted, considering the indications and doses prescribed in the first 24 hours after admission for the 574 patients who did not have contraindications. For these patients, compliance between the need for chemoprophylaxis indicated by the risk stratification process and the chemoprophylaxis actually prescribed was observed in 438 (76%) of the cases analyzed and was significantly more prevalent (p < 0.0001) among low-risk clinical patients (195; 91%), low-risk surgical patients (41; 98%), and high-risk surgical patients (94; 86%) than in the other subsets. Notwithstanding, 20 clinical patients (9%) and one low-risk surgical patient (2%) were unnecessarily prescribed chemoprophylaxis, rather than only being prescribed early mobilization, while 15 high-risk surgical patients (14%) were not prescribed it. Among the high-risk clinical patients and the moderate-risk surgical patients, compliance between the need for chemoprophylaxis and its administration was observed in 65% (91) 25% (17), respectively, which are also significantly different in relation to the other subsets. 

**Table 6:** Compliance with recommendations for administration of chemoprophylaxis and daily dose prescribed during first 24 hours after admission of clinical and surgical patients, according to stratification by risk of venous thromboembolism.

**Risk stratification**	**n**	**Compliance of chemoprophylaxis administration with indication** **(need vs. administration)**		**Compliance of daily chemoprophylaxis dose prescribed**		
		**n**	**%**	**n**	**% (indication vs. dose)**	**% (dose vs. need)**
Clinical patients						
Low risk	215	195	91	--	--	--
High risk	140	91	65	76	84%	54%
Subtotal	355	286	81	76	84%	54%
Surgical patients						
Very low risk/low risk	42	41	98	--	--	--
Moderate risk	68	17	25	3	18%	4%
High risk	109	94	86	93	99%	85%
Subtotal	219	152	69	96	86%	54%
Total	574	438	76	172	85%	54%

p < 0.0001 low-risk clinical patients vs. high-risk clinical patients; p < 0.0001 high-risk clinical patients vs. high-risk surgical patients; p < 0.0001 low-risk surgical patients vs. moderate-risk surgical patients;

p < 0.0001 moderate-risk surgical patients vs. high-risk surgical patients.

 With regard to non-prescription of chemoprophylaxis for patients who needed it during the first 24 hours after admission, it was observed that 35% (49) of the clinical patients and 37% (66) of the surgical patients were not prescribed chemoprophylaxis despite needing it. There was no significant difference between these two subsets in terms of non-compliance with thromboprophylaxis recommendations (p = 0.76). 

 The results of analysis of the chemoprophylaxis dosages prescribed for the subset of patients who were given it during the first 24 hours after admission (indications vs. actual dose) revealed compliance in 84% (76) of the high-risk clinical patients and 99% (93) of the high-risk surgical patients. However, management was only compliant with recommendations in 18% (3) of the moderate-risk surgical patients, revealing significant differences between these subsets (p < 0.0001). 

 The overall chemoprophylaxis compliance assessment for patients at moderate and high risk of VTE took into account the chemoprophylaxis doses prescribed and the total number of patients who required it within each of these risk strata, irrespective of whether they were or were not given it (dose vs. need). The result of this calculation was to reduce the chemoprophylaxis compliance rates to just 54% (76) of the total subset of 140 high-risk clinical patients and 4% (3) of the total subset of 68 moderate-risk surgical patients, although it remained at 85% (93) of the total of 109 high-risk surgical patients (p < 0.0001). 

**Figure 2:**
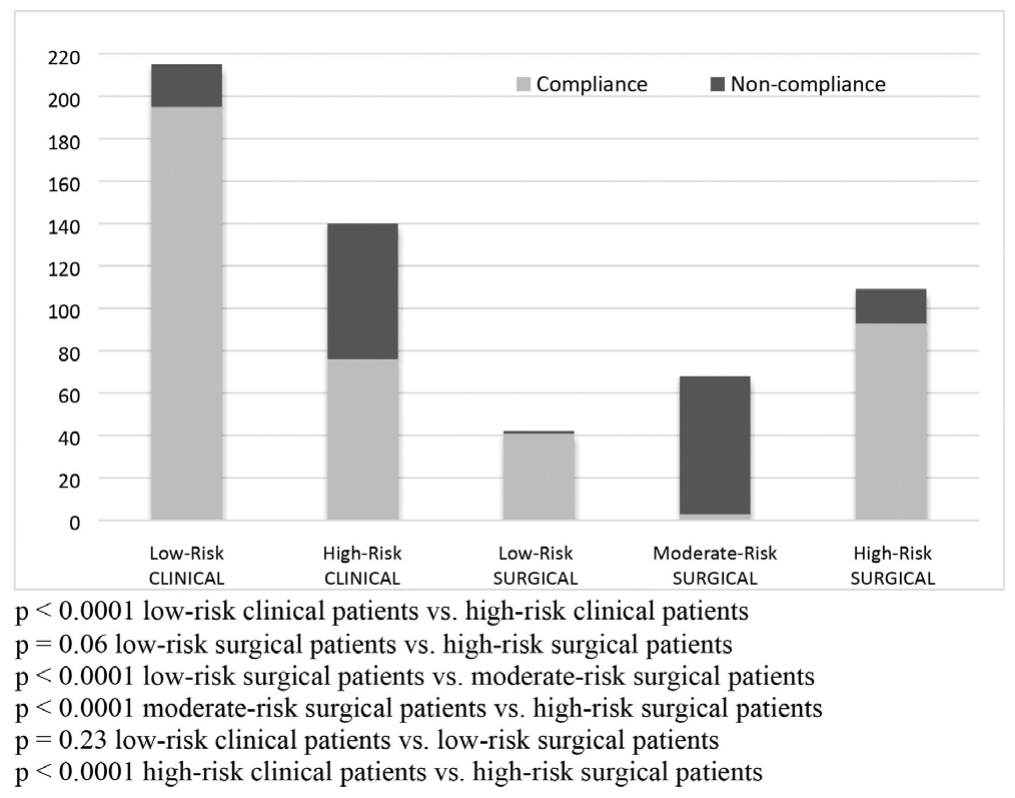
Results of assessment of compliance of thromboprophylaxis administered to clinical and surgical patients during first 24 hours after admission, according to VTE risk stratification.

Figure 2 illustrates and confirms the results reported up to this point, showing that thromboprophylaxis was more often administered adequately to low-risk clinical and surgical patients and to high-risk surgical patients, whose rates of prophylaxis compliance did not differ significantly from each other (p > 0.05), but were significantly different from the other subsets. In contrast, prophylaxis was underutilized for high-risk clinical patients and moderate-risk surgical patients, whose prophylaxis compliance rates were significantly different from those of the other subsets (p < 0.0001). 

 There were also certain specific situations worthy of note. A group of 57 obese patients admitted for bariatric surgery were stratified as high risk and accounted for 50% of the high-risk patients. There were all given 40 mg enoxaparin once a day, mechanical prophylaxis (elastic stockings), lower limb motor physiotherapy, and early mobilization within 24 hours of admission. There were also 23 patients with renal failure (four high-risk surgical patients – 4%; and 19 high-risk clinical patients – 14%), whose creatinine clearance rates exceeded 30 mL/min and so chemoprophylaxis dose adjustment was not recommended. 

 Furthermore, it was observed that although the institution systematically attaches its institutional VTE prophylaxis protocol to all patient charts at admission, this document was only completed for 32 (5%) of the patients during the study period. 

## DISCUSSION

 The profile of the patients admitted, in terms of the proportions of clinical (62%) and surgical patients (38%), was no different to what has been observed at other general hospitals, 10 ^,^ 17 although patient risk stratification profiles do vary greatly at different institutions. Coexistence of several different guidelines, differences between patient profiles and, possibly, non-uniform analysis methods may be responsible for differences in the results reported by different authors. It should also be considered that this study only analyzed conduct during the first 24 hours after admission of patients. It was nevertheless possible to observe that there was a consistent and considerable proportion of patients who did benefit from chemoprophylaxis; in this case, 81% of the surgical patients and 42% of the clinical patients. This scenario justifies carrying out studies to assess compliance of hospital conduct with relation to thromboprophylaxis. 

 Stratification of patients by risk category is considered the most appropriate tool for taking decisions on the prophylactic measures to be employed. Each patient’s potential risk of VTE should therefore be calculated at the time of hospital admission and thromboprophylaxis should be initiated as soon as possible. 7 ^,^ 18 The Padua and Caprini scores proved to be useful for this purpose and easy to use, since they attribute scores for different risk factors and help to illustrate how patients with the same number of risk factors may nevertheless be allocated to different VTE risk strata. They also demonstrate the importance of restricted mobility among high-risk clinical patients, the scale of surgery among patients in the 41-60 years age group, and obesity and confinement to bed among moderate and high-risk surgical patients. 

 The mean number of risk factors observed in patients in all of the different study population strata confirms the ACCP’s VTE prevention statement that almost all hospitalized patients have at least one risk factor for VTE. 5 The low prevalence of patients with contraindications against chemoprophylaxis is also similar to other authors’ results 17 and shows that administration is safe. 

 Busato et al. 19 recommend use of lower limb motor physiotherapy in cases in which chemoprophylaxis is contraindicated. They advise its use for all patients with any level of VTE risk, both in cases with contraindications against anticoagulants and as an adjuvant to pharmacological treatment. Therefore, although it is not strictly recommended as a mechanical thromboprophylaxis method by guidelines on the subject, use of motor physiotherapy was defined as compliant, since it is compatible with the situation in the majority of Brazilian hospitals. 

 It was found that there was no difference between rates of thromboprophylaxis noncompliance in clinical and surgical patients, as was also observed by Fuzinatto et al. 17 and Carneiro et al. 20 Underutilization of chemoprophylaxis was the most common reason for noncompliance among both clinical and surgical patients. This has also been observed by other authors, 10 ^,^ 17 ^,^ 20 ^,^ 21 who documented underutilization among patients at high risk of development of VTE and its complications, reporting evidence of noncompliance between what is recommended by thromboprophylaxis protocols and what actually takes place in hospitals. 

 In the analysis by risk strata, it was repeatedly observed that both clinical and surgical low-risk patients and high-risk surgical patients were better identified and managed during the first 24 hours after hospital admission. There were no significant differences in thromboprophylaxis compliance between these groups (p > 0.05), but its prevalence was significantly higher than in all other subsets (p < 0.0001). However, comparison of high-risk patients in isolation revealed that the prevalence of compliance among high-risk surgical patients (85%) was significantly higher than for high-risk clinical patients (54%) (p < 0.0001). The highest noncompliance prevalence was observed among moderate-risk surgical patients (4%), both in terms of identification of a need for prescription of chemoprophylaxis and in terms of the daily dose prescribed (p < 0.0001). These patients were more likely not to be identified as at risk of VTE and, when they were identified as at risk, they were generally given similar daily doses to the high-risk surgical patients, so they were potentially more likely to be exposed to VTE in the first case or exposed to bleeding in the second. Data showing similar failures, especially of prophylaxis among patients at moderate risk, were also reported by Dhamnaskar et al. 22 and Pereira et al., 10 although the latter also observed that physicians treating surgical patients prescribed prophylaxis less frequently that the physicians of clinical patients. It could therefore be inferred that the profile of factors found among high-risk surgical patients, such as major surgery and obesity, were more likely to be recognized by the prescribing surgeon than reduced mobility and advanced age, found among the high-risk clinical patients, or being confined to bed for short periods among moderate-risk surgical patients. These observations underscore the multidisciplinary character of VTE prevention. 

 In certain specific situations, such as bariatric surgery, it has been suggested that these and other obese patients may need higher doses of anticoagulants, since the greater volume of adipose tissue may interfere with absorption of pharmaceuticals administered subcutaneously. As routine management, the majority of services use chemical prophylaxis, i.e., they employ subcutaneous administration of unfractionated heparin (UFH) at 10,000 to 15,000 units/day, split across two or three doses, or low molecular weight heparin (LMWH) in two doses per day (30 mg or 40 mg enoxaparin). Since coexistence of multiple risk factors confers an even higher risk of thromboembolic events in these patients, most services use a combination of physical and chemical measures to increase the efficacy of VTE prevention. 23 In this study, it was observed that all 57 bariatric surgery patients were given chemoprophylaxis and the mechanical method, showing that the institution’s conduct is in compliance with the options described in the literature. 

 With relation to the patients with renal failure, it was observed that management was in line with ACCP recommendations, which advise adjusting posology for patients with creatinine clearance < 30 mL/min, since there is increased exposure to the medication and risk of bleeding due to factor Xa build up. The normal dose can still be prescribed in cases of moderate and mild renal insufficiency. 24

 Although it is systematically attached to the patient’s medical charts at admission, the institution’s protocol was only completed for 5% of recently-admitted patients, so the institution is unaware of its clients’ VTE risk profiles and is less able to take clear and uniform measures with relation to the relationship between risk stratification of patients and the thromboprophylaxis adopted. This confirms findings reported by other authors, who have shown that passive distribution of protocols and merely announcing thromboprophylactic strategies have a low probability of success. 5 ^,^ 12 ^,^ 25 These authors consider that implementation of educational measures combined with other strategies for improving quality – setting up multidisciplinary commissions, audits, and real time feedback on the recommendations of protocols – and technological informational initiatives, such as computerized alerts and mandatory clinical decision-making support systems appear to be more effective options for promoting implementation of best prophylactic practices and preventing patient harm from VTE. 

 Since this is a cross- sectional study that only investigated conduct on the first day of new admissions, its results cannot be extrapolated to adequacy of thromboprophylaxis throughout the entire period of these patients’ time in hospital. However, they nevertheless indicate a need for effective programs that are designed to ensure patient safety in relation to VTE in the first hours after admission. 

## CONCLUSIONS

 The stratification process revealed that 57% of all recently-admitted patients had indications for chemoprophylaxis during the first 24 hours after admission: 42% of the clinical patients and 81% of the surgical patients. However, the results for compliance of the prophylaxis provided confirm reports in the literature, showing that there is underutilization of chemical VTE prophylaxis, both for clinical patients and for surgical patients. The most important findings were the rates for high-risk clinical patients and moderate-risk surgical patients, since just 54% and 4%, respectively, were given the appropriate chemoprophylaxis during the period analyzed. There is a need to improve patient safety in relation to VTE during the first hours after admission. 
