# Supplementary material for: Evaluation of deep vein thrombosis prophylaxis in a general hospital
Source: J Vasc Bras. 2018 Jul-Sep;17(3):184–92. doi: 10.1590/1677-5449.007017 (PMC6326129; doi:10.1590/1677-5449.007017)
Supplement: Translation

# Translation: Avaliação da profilaxia da trombose venosa profunda em um hospital geral

**Authors:** Fátima Cristiane Lopes Goularte Farhat, Hellen Caroliny Torres Gregório, Rafaela Durrer Parolina de Carvalho

**Affiliations:** 1 Universidade Metodista de Piracicaba – UNIMEP, Faculdade de Ciências da Saúde, Curso de Farmácia, Piracicaba, SP, Brasil.; 2 Hospital Irmandade da Santa Casa de Misericórdia de Piracicaba, Serviço de Farmácia, Piracicaba, SP, Brasil.; 3 Universidade Estadual de Campinas – UNICAMP, Faculdade de Odontologia de Piracicaba – FOP, Piracicaba, SP, Brasil.

**Keywords:** tromboembolismo venoso, quimioprofilaxia, heparina, hospital

## Abstract

**Contexto:**

O tromboembolismo venoso (TEV) representa uma preocupação crescente nas instituições hospitalares, tem grande impacto sobre a morbimortalidade em pacientes clínicos e cirúrgicos, e é a principal causa de morte evitável hospitalar. Embora existam modelos de avaliação de risco para pacientes hospitalizados, a profilaxia ainda é subutilizada ou é feita de forma incorreta.

**Objetivos:**

Avaliar o perfil de risco para TEV de pacientes clínicos e cirúrgicos recém-internados, bem como as medidas tromboprofiláticas aplicadas nas primeiras 24 horas de internação.

**Métodos:**

Este estudo transversal foi realizado em um hospital geral de grande porte do interior do estado de São Paulo entre março e julho de 2015. Os escores de Pádua e Caprini foram utilizados para estratificação de risco dos pacientes clínicos e cirúrgicos, respectivamente, enquanto a análise das medidas tromboprofiláticas baseou-se nas recomendações do 8º e 9º Consenso do *American College of Chest Physicians*.

**Resultados:**

Foram analisados 592 pacientes (62% clínicos e 38% cirúrgicos). A estratificação de risco revelou necessidade de quimioprofilaxia em 42% dos pacientes clínicos e 81% dos cirúrgicos (51% de alto risco e 30% de moderado risco). Por outro lado, receberam profilaxia adequada nas primeiras 24 horas de internação 54% dos pacientes clínicos de alto risco, 85% dos cirúrgicos de alto risco e 4% dos cirúrgicos de moderado risco, todos sem contraindicação.

**Conclusões:**

Há necessidade de aprimoramento da segurança do paciente em relação ao TEV já nas primeiras horas de internação. Existe uma subutilização da quimioprofilaxia especialmente nos pacientes clínicos de alto risco e cirúrgicos de moderado risco.

## INTRODUÇÃO

 A trombose venosa profunda (TVP) resulta da formação de trombos em veias profundas. É mais comum em membros inferiores, mas pode acometer a veia cava, as veias jugulares internas e os membros superiores. Os trombos podem causar oclusão parcial ou total do sistema venoso profundo, e a complicação imediata mais grave é a embolia pulmonar (EP), que ocorre após o desprendimento de um trombo e a obstrução do fluxo sanguíneo na artéria pulmonar, com consequentes eventos cardiorrespiratórios. 1 ^,^ 2

 O tromboembolismo venoso (TEV) compreende essas duas doenças relacionadas, TVP e EP. Episódios assintomáticos ou clinicamente aparentes em pacientes hospitalizados podem estar associados à mortalidade. Assim, o TEV é considerado a principal causa de óbito evitável em ambiente hospitalar. 1 ^,^ 3 ^-^ 5 Trata-se de uma doença comum em pacientes hospitalizados, que pode aparecer como complicação de outras afecções clínicas ou cirúrgicas, mas também pode ocorrer de forma espontânea em pessoas aparentemente sadias. 6 Segundo o 8º Consenso do *American College of Chest Physicians* (ACCP) sobre prevenção do TEV, quase todos os pacientes hospitalizados têm pelo menos um fator de risco para o desenvolvimento do TEV, e cerca de 40% têm três ou mais. A tromboprofilaxia é a estratégia inicial para melhorar a segurança de pacientes hospitalizados. 5

 Estudos comprovam que a tromboprofilaxia é efetiva e segura. Medidas como deambulação precoce, meias elásticas de compressão graduada, compressão pneumática intermitente e uso de anticoagulantes devem ser adotadas racionalmente após a adequada estratificação de risco dos pacientes, para não os expor a medidas desnecessárias. Também é importante não deixar de fazê-las nos pacientes com indicação. 4 ^-^ 9

 Por outro lado, observa-se uma baixa aderência à prescrição de tromboprofilaxia. Além disso, quando realizada, costuma ser feita de forma incorreta mesmo com a disponibilidade de protocolos aos profissionais da área da saúde. 6 ^,^ 10 ^-^ 12

 Sendo assim, este trabalho visa avaliar o perfil de risco para o TEV de pacientes clínicos e cirúrgicos recém-internados, bem como as medidas tromboprofiláticas aplicadas nas primeiras 24 horas de internação. 

## MÉTODOS

 Este estudo transversal e descritivo foi realizado em um hospital geral de grande porte do interior do estado de São Paulo. Foram analisadas as primeiras 24 horas de internação de pacientes clínicos e cirúrgicos com mais de 18 anos, recém-internados e que permaneceram na instituição por mais de 24 horas, entre os meses de março e julho de 2015. Foram excluídos pacientes pediátricos, gestantes e puérperas, pacientes já em tratamento de episódios trombóticos, além daqueles cujas informações não estavam disponíveis após três tentativas consecutivas de avaliação em ao menos dois dias diferentes. Pacientes internados como cirúrgicos, mas que não realizaram o procedimento cirúrgico nas primeiras 48 horas de internação foram reclassificados e avaliados como clínicos. 

 Para estratificação de risco e avaliação de tromboprofilaxia, foi desenvolvido um fluxograma ( Figura 1 ) baseado nas recomendações do ACCP sobre prevenção do TEV. 5 ^,^ 13 ^,^ 14 Foram adotados os escores de Pádua 15 para estratificação de risco dos pacientes clínicos e de Caprini 16 para pacientes cirúrgicos, bem como possíveis contraindicações e condutas em situações especiais como pacientes obesos (índice de massa corporal, IMC ≥ 30) e insuficiência renal. Informações sobre fatores de risco para TEV presentes nas primeiras 24 horas de internação dos pacientes, medidas tromboprofiláticas adotadas, contraindicações para quimioprofilaxia e situações especiais foram coletadas junto à equipe de saúde e no prontuário do paciente. 

**Figura 1:** Fluxograma utilizado para estratificação de risco para TEV e avaliação da tromboprofilaxia aplicada aos pacientes internados.

 A Tabela 1 mostra a relação entre os escores adotados e a estratificação de risco dos pacientes clínicos e cirúrgicos, bem como a tromboprofilaxia recomendada pelo ACCP. Pacientes cirúrgicos de muito baixo e baixo risco foram agrupados por apresentarem a mesma recomendação profilática. 

**Tabela 1:** Estratificação de risco segundo os escores de Pádua e Caprini e tromboprofilaxia recomendada pelo *American College of Chest Physicians* .

**Estratificação de risco**		**Tromboprofilaxia**
		Escore de Pádua – Pacientes clínicos
< 4 pontos	Baixo risco	Deambulação precoce
≥ 4 pontos	Alto risco	HNF: 5.000 UI 8/8h HBPM: 40 mg 1x/dia Profilaxia mecânica quando houver contraindicação da quimioprofilaxia e reconsiderar quando o risco de sangramento diminuir
		Escore de Caprini – Pacientes cirúrgicos
0 ponto	Muito baixo risco	Deambulação precoce
1-2 pontos	Baixo risco	Deambulação precoce
3-4 pontos	Moderado risco	HNF: 5.000 UI 12/12h HBPM: 20 mg 1x/dia Profilaxia mecânica quando houver contraindicação da quimioprofilaxia e reconsiderar quando o risco de sangramento diminuir
≥ 5 pontos	Alto risco	HBPM: 40 mg 1x/dia Profilaxia mecânica quando houver contraindicação da quimioprofilaxia e reconsiderar quando o risco de sangramento diminuir

HBPM, heparina de baixo peso molecular; HNF, heparina não fracionada

 A partir da estratificação de risco do paciente, a conformidade da tromboprofilaxia foi avaliada de acordo com dois quesitos: indicação e dose diária da quimioprofilaxia. Para esse fim, considerou-se conformidade se a quimioprofilaxia foi prescrita para os casos necessários e, posteriormente, se a dose diária prescrita estava adequada aos pacientes que a receberam, além de sua não prescrição para os casos em que não era necessária. Por outro lado, considerou-se não conformidade a prescrição de quimioprofilaxia para os casos em que não era necessária e a falta desta, ou sua realização em dose inadequada para os casos em que era necessária. 

 Os resultados foram organizados em tabelas de contingência 2x2 e avaliados com o auxílio do pacote estatístico BioEstat 5.3, com aplicação do teste do qui-quadrado. O nível de significância adotado foi p < 0,05. 

 Este estudo foi aprovado pelo Comitê de Ética em Pesquisa da Universidade Metodista de Piracicaba/UNIMEP (protocolo nº 36/2014) e foi realizado no Hospital dos Fornecedores de Cana de Piracicaba (HFCP). 

## RESULTADOS

 Foram analisados 592 pacientes em suas primeiras 24 horas de internação, sendo 369 (62%) pacientes clínicos e 223 (38%) pacientes cirúrgicos. A prevalência dos fatores de risco para TEV e seus níveis de importância nas primeiras horas de internação dos pacientes clínicos e cirúrgicos são revelados nas Tabelas 2 e 3 , respectivamente. Pode-se observar que os pacientes clínicos somaram 594 fatores de risco (média de 1,6/paciente), enquanto os cirúrgicos, 575 (média de 2,6/paciente). 

**Tabela 2:** Fatores de risco encontrados nos pacientes clínicos, segundo o escore de Pádua.

**Fatores de risco**	**Escore**	**n**	**% dos pacientes**
Mobilidade reduzida	3	214	58
Idade avançada (≥ 70 anos)	1	150	41
Infecções e/ou doenças reumatológicas	1	87	24
Insuficiência cardíaca e/ou respiratória	1	57	15
Obesidade (IMC ≥ 30)	1	38	10
Câncer em atividade	3	25	7
Infarto agudo do miocárdio ou acidente vascular cerebral	1	12	3
Trauma ou cirurgia recente (último mês)	2	8	2
Terapia hormonal atual	1	2	0,5
História prévia de TEV (excluindo trombose venosa superficial)	3	1	0,3
Trombofilia conhecida	3	0	0
Total		594	

IMC, índice de massa corporal; TEV, tromboembolismo venoso.

**Tabela 3:** Fatores de risco encontrados nos pacientes cirúrgicos, segundo o escore de Caprini.

**Fatores de risco**	**Escore**	**n**	**% dos pacientes**
Cirurgia de grande porte (> 60 min)	2	134	60
Cirurgia de pequeno porte	1	89	40
Idade 41-60	1	87	39
Obesidade (IMC ≥ 30)	1	73	33
Restrição ao leito	1	55	25
Idade 61-74	2	41	18
Restrição ao leito (> 72 horas)	2	34	15
Idade ≥ 75 anos	3	22	10
Câncer	2	9	4
Cateter venoso central	2	9	4
Edema de MMII	1	5	2
Artroplastia de joelho ou quadril	5	4	2
Artroscopia	2	2	1
Politrauma	5	2	1
IAM	1	2	1
Varizes	1	2	1
História familiar de TEV	3	1	0,4
Cirurgia de grande porte prévia (< 1 mês)	1	1	0,4
DPOC	1	1	0,4
Sepse (< 1 mês)	1	1	0,4
Contraceptivo oral/terapia de reposição hormonal	1	1	0,4
Total		575	

DPOC, doença pulmonar obstrutiva crônica; IAM, infarto agudo do miocárdio; IMC, índice de massa corporal; MMII, membros inferiores; TEV, tromboembolismo venoso.

 Já a Tabela 4 demonstra o perfil dos pacientes analisados em relação à estratificação de risco para o TEV, número médio de fatores de risco/paciente, fatores de risco mais prevalentes em cada grupo, bem como as recomendações de tromboprofilaxia pelo ACCP 5 ^,^ 13 ^,^ 14 . Observa-se que a elevação do risco para TEV é acompanhada pela elevação no número médio de fatores de risco associados nos pacientes, além da prevalência de fatores com maior pontuação nos escores correspondentes. 

**Tabela 4:** Perfil dos pacientes clínicos e cirúrgicos em relação à estratificação de risco para tromboembolismo venoso, número médio de fatores de risco/paciente, fatores de risco mais prevalentes e profilaxia preconizada pelo *American College of Chest Physicians*.

**Estratificação de risco**	**n (%)**	**Nº médio de fatores de risco/paciente (mín.-máx.)**	**Fatores de risco mais prevalentes** **nos pacientes analisados**	**Tromboprofilaxia preconizada**
Pacientes clínicos	369	1,6		
Baixo risco	215 (58%)	0,9 (0-3)	Idade > 70	Deambulação precoce
Alto risco	154 (42%)	2,6 (2-4)	Mobilidade reduzida, idade > 70 e infecções ou doenças reumatológicas em atividade	HNF: 5.000 UI 8/8h HBPM: 40 mg 1x/dia
Pacientes cirúrgicos	223	2,6		
Muito baixo/baixo risco	42 (19%)	1,6 (1-2)	Cirurgia de pequeno porte, idade 41-60	Deambulação precoce
Moderado risco	68 (30%)	2,4 (1-3)	Cirurgia de pequeno ou grande porte, idade 41-60, restrição ao leito	HNF: 5.000 UI 12/12h HBPM: 20 mg 1x/dia
Alto risco	113 (51%)	3,1 (2-5)	Cirurgia de grande porte, obesidade, idade 41-60 anos	HBPM: 40 mg 1x/dia

HBPM, heparina de baixo peso molecular; HNF, heparina não fracionada.

 O processo de estratificação identificou 154 (42%) pacientes clínicos de alto risco, além de, respectivamente, 68 (30%) e 113 (51%) pacientes cirúrgicos de moderado e alto risco para TEV. Sendo assim, pode-se observar que 335 (57%) pacientes do total analisado apresentavam indicação para uso da quimioprofilaxia já em suas primeiras horas de internação, sendo 42% (154) dos clínicos e 81% (181) dos cirúrgicos. 

 Por outro lado, apenas 18 (3%) pacientes apresentaram evidências de contraindicação para a quimioprofilaxia, sendo 14 clínicos (3,8%) e quatro cirúrgicos (1,8%). Os motivos identificados estão descritos na Tabela 5 . Para a totalidade desses casos, observou-se a prescrição de fisioterapia motora de membros inferiores, realizada de 2 a 3 vezes por dia, como provável medida tromboprofilática. 

**Tabela 5:** Contraindicação para quimioprofilaxia nos pacientes analisados.

**Contraindicação**	**n**	**%**
Uso de anticoagulante	11	61
Sangramento ativo	4	22
HAS não controlada (> 180 x 110 mmHg)	2	11
Plaquetopenia	1	6
Total	18	100

HAS, hipertensão arterial sistêmica.

 Na sequência, a Tabela 6 demonstra a conformidade da tromboprofilaxia adotada, considerando-se indicação e dose prescrita nas primeiras 24 horas de internação dos 574 pacientes que não apresentavam contraindicação. Do total de pacientes, pode-se observar conformidade entre a necessidade preconizada pela estratificação de risco e a indicação de quimioprofilaxia em 438 (76%) casos analisados, sendo significantemente mais prevalente (p < 0,0001) entre os pacientes clínicos de baixo risco (195; 91%), cirúrgicos de baixo risco (41; 98%) e cirúrgicos de alto risco (94; 86%), em relação aos demais grupos. Mesmo assim, 20 pacientes clínicos (9%) e um paciente cirúrgico de baixo risco (2%) foram submetidos ao uso desnecessário de quimioprofilaxia, em vez de somente deambulação precoce, enquanto 15 pacientes cirúrgicos de alto risco (14%) deixaram de recebê-la. Entre os pacientes clínicos de alto risco e os cirúrgicos de moderado risco, por outro lado, a conformidade entre necessidade de quimioprofilaxia e sua indicação ocorreu em 65% (91) e 25% (17) dos pacientes, respectivamente, revelando diferença também significante em relação aos demais grupos. 

**Tabela 6:** Conformidade quanto à indicação e à dose diária da quimioprofilaxia prescrita nas primeiras 24 horas de internação de pacientes clínicos e cirúrgicos, conforme a estratificação de risco para tromboembolismo venoso.

**Estratificação de risco**	**n**	**Conformidade da quimioprofilaxia quanto à indicação (necessidade *vs.* indicação)**		**Conformidade da quimioprofilaxia quanto à dose diária prescrita**		
		**n**	**%**	**n**	**% (indicação *vs.* dose)**	**% (dose *vs.* necessidade)**
Pacientes clínicos						
Baixo risco	215	195	91	--	--	--
Alto risco	140	91	65	76	84%	54%
Subtotal	355	286	81	76	84%	54%
Pacientes cirúrgicos						
Muito baixo/baixo risco	42	41	98	--	--	--
Moderado risco	68	17	25	3	18%	4%
Alto risco	109	94	86	93	99%	85%
Subtotal	219	152	69	96	86%	54%
Total	574	438	76	172	85%	54%

p < 0,0001 entre pacientes clínicos de baixo e alto risco; p < 0,0001 entre pacientes clínicos e cirúrgicos de alto risco; p < 0,0001 entre pacientes cirúrgicos de baixo e moderado risco; p < 0,0001 entre pacientes cirúrgicos de moderado e alto risco.

 Quando considerada a não prescrição da quimioprofilaxia para o conjunto de pacientes que necessitavam já nas primeiras 24 horas de sua internação, observou-se que 35% (49) dos pacientes clínicos e 37% (66) dos cirúrgicos deixaram de recebê-la. Não houve diferença significante entre esses dois grupos no quesito não conformidade na indicação da tromboprofilaxia (p = 0,76). 

 Ao se avaliar a dose da quimioprofilaxia prescrita ao conjunto de pacientes que a recebeu durante as primeiras 24 horas de internação (indicação *vs.* dose), os resultados revelaram conformidade em 84% (76) dos pacientes clínicos de alto risco e 99% (93) dos cirúrgicos de alto risco. Porém, ocorreu em apenas 18% (3) dos pacientes cirúrgicos de moderado risco, revelando diferenças significantes entre esses grupos (p < 0,0001). 

 A conformidade final da quimioprofilaxia para os pacientes de moderado a alto risco de TEV levou em conta a dose prescrita da quimioprofilaxia e o total de pacientes com necessidade em cada um desses estratos de risco, independentemente de tê-la recebido ou não (dose *vs.* necessidade). Isso resultou em redução da conformidade da quimioprofilaxia para apenas 54% (76) do total de 140 pacientes clínicos de alto risco e 4% (3) do total de 68 pacientes cirúrgicos de moderado risco, enquanto manteve-se em 85% (93) do total de 109 pacientes cirúrgicos de alto risco (p < 0,0001). 

 A Figura 2 demonstra e corrobora os resultados obtidos até o momento, de que a tromboprofilaxia foi mais adequadamente aplicada nos pacientes clínicos e cirúrgicos de baixo risco, bem como nos pacientes cirúrgicos de alto risco, os quais não apresentam diferença significante entre si na conformidade da profilaxia (p > 0,05), embora apresentem em relação aos demais grupos. Por outro lado, houve subutilização da profilaxia nos pacientes clínicos de alto risco e cirúrgicos de moderado risco, os quais apresentaram diferenças significantes na conformidade da profilaxia em relação aos demais grupos (p < 0,0001). 

**Figura 2:** Avaliação da conformidade da tromboprofilaxia aplicada a pacientes clínicos e cirúrgicos nas primeiras 24 horas de internação, de acordo com a estratificação de risco dos pacientes.

 Cabe ressaltar a presença de situações especiais, representadas por 57 pacientes obesos em cirurgia bariátrica, os quais foram estratificados como de alto risco e corresponderam a 50% desses pacientes. Todos receberam enoxaparina 40 mg 1x/dia, associada à profilaxia mecânica (meias elásticas), fisioterapia motora de membros inferiores e deambulação precoce nas primeiras 24 horas de internação. Além disso, foram identificados 23 pacientes com insuficiência renal (quatro pacientes cirúrgicos de alto risco – 4%; e 19 clínicos de alto risco – 14%), nos quais o *clearance* de creatinina era superior a 30 mL/min e não era recomendado, portanto, ajuste de dose da quimioprofilaxia. 

 Observou-se que, embora a instituição sistematicamente anexasse seu protocolo institucional para profilaxia do TEV a todos os prontuários no ato da internação dos pacientes, apenas 32 (5%) desses tiveram o documento preenchido durante o período de realização da pesquisa. 

## DISCUSSÃO

 O perfil de pacientes internados, entre clínicos (62%) e cirúrgicos (38%), não difere do encontrado em outros hospitais gerais 10 ^,^ 17 , embora a estratificação de risco dos pacientes nas diferentes instituições apresente grande variação. A coexistência de várias diretrizes, as diferenças entre os perfis de pacientes e uma possível não uniformidade nas análises podem acarretar resultados diversos entre os diferentes autores. Deve-se considerar também que o presente trabalho avaliou apenas as primeiras 24 horas de internação dos pacientes. Apesar disso, é possível observar a presença constante de um grupo expressivo de pacientes que se beneficiariam da quimioprofilaxia; neste caso, 81% dos pacientes cirúrgicos e 42% dos clínicos. Tal cenário justifica a realização de trabalhos que avaliem a conformidade das condutas hospitalares com relação à tromboprofilaxia. 

 A estratificação dos pacientes em categorias de risco é a ferramenta considerada mais adequada para a tomada de decisão quanto à medida profilática a ser adotada. Portanto, o risco potencial de TEV de cada paciente deve ser calculado já na admissão hospitalar, e a tromboprofilaxia deve ser iniciada o mais breve possível 7 ^,^ 18 . Nesse sentido, os escores de Pádua e Caprini revelaram-se úteis e fáceis de utilizar, uma vez que conferem pontuações para os diferentes fatores de risco e auxiliam na compreensão de que pacientes com o mesmo número de fatores podem ser estratificados em diferentes níveis de risco para o TEV. Foi possível perceber, assim, a importância da mobilidade reduzida entre os pacientes clínicos de alto risco e, por outro lado, o porte cirúrgico associado à idade 41-60 anos, restrição ao leito e obesidade entre os pacientes cirúrgicos de moderado a alto risco. 

 A média de fatores de risco presente nos diferentes estratos da população estudada corrobora as afirmações realizadas pelo ACCP sobre prevenção do TEV, de que quase todos os pacientes hospitalizados têm pelo menos um fator de risco para TEV 5 . Da mesma forma, a baixa prevalência de pacientes com contraindicação para quimioprofilaxia está em consonância com outros autores 17 e assegura sua aplicação. 

 A utilização de fisioterapia motora de membros inferiores nos casos em que a quimioprofilaxia é contraindicada é defendida por Busato et al. 19 . Segundo os autores, ela é recomendada para todos os pacientes com diferentes riscos de TEV, podendo atuar nos casos com contraindicação ao uso de anticoagulantes ou como adjuvante à terapia farmacológica. Sendo assim, mesmo não claramente preconizada como tromboprofilaxia mecânica pelas diretrizes sobre o assunto, a aplicação da fisioterapia motora foi considerada como conformidade por condizer com a realidade da maioria dos hospitais brasileiros. 

 Constatou-se que não houve diferença quando comparadas as taxas de inadequação da tromboprofilaxia entre os pacientes clínicos e cirúrgicos, assim como já observado por Fuzinatto et al. 17 e Carneiro et al. 20 . A subutilização da quimioprofilaxia foi a principal causa de não conformidade tanto nos pacientes clínicos como nos cirúrgicos. Isso foi apontado também por outros autores 10 ^,^ 17 ^,^ 20 ^,^ 21 , os quais demonstraram subutilização mesmo em pacientes com alto risco para o desenvolvimento do TEV e suas complicações, evidenciando a não conformidade entre o preconizado em protocolos de tromboprofilaxia e a realidade encontrada nos hospitais. 

 Na análise por estratos de risco, observou-se repetidamente que os pacientes de baixo risco, tanto clínicos como cirúrgicos, e os de alto risco cirúrgico foram melhor identificados e conduzidos já nas suas primeiras 24 horas de internação hospitalar. Não houve diferenças significantes quanto à conformidade da tromboprofilaxia entre esses grupos (p > 0,05), mas ela foi significantemente mais prevalente em relação aos demais grupos (p < 0,0001). Por outro lado, quando comparados somente os pacientes de alto risco, observou-se que os pacientes cirúrgicos apresentaram maior prevalência de conformidade (85%) do que os clínicos (54%) (p < 0,0001). Os pacientes cirúrgicos de moderado risco representaram o grupo com maior prevalência de não conformidade (4%), tanto na identificação da necessidade de prescrição da profilaxia química quanto na dose diária a ser prescrita (p < 0,0001). Tais pacientes, além de serem insuficientemente identificados como pacientes de risco para o TEV, quando o eram, geralmente receberam doses diárias semelhantes aos pacientes cirúrgicos de alto risco, ficando, portanto, potencialmente mais expostos ao TEV ou, por outro lado, mais expostos a sangramentos. Informações sobre falhas, especialmente na profilaxia de pacientes de moderado risco, também foram encontradas por Dhamnaskar et al. 22 e por Pereira et al. 10 , embora os últimos tenham observado que os médicos cirurgiões prescreveram a profilaxia menos frequentemente do que os médicos clínicos. Nesse sentido, pode-se inferir que o perfil dos fatores encontrados nos pacientes cirúrgicos de alto risco, como cirurgia de grande porte e obesidade, foi mais facilmente reconhecido pelo prescritor do que mobilidade reduzida e idade avançada, presentes nos pacientes clínicos de alto risco, bem como a restrição ao leito por curto prazo entre os pacientes cirúrgicos de moderado risco. Tais situações reforçam o caráter multiprofissional envolvido na prevenção do TEV. 

 Em situações especiais, como de cirurgia bariátrica, sugere-se que esses e outros pacientes obesos podem necessitar de doses mais elevadas de anticoagulantes, uma vez que a maior quantidade de tecido adiposo poderia atrapalhar a absorção de fármacos administrados de forma subcutânea. Dessa forma, rotineiramente, a maioria dos serviços opta pela profilaxia química, ou seja, utilizam a aplicação subcutânea de heparina não fracionada (HNF) 10.000 a 15.000 unidades/dia dividida em duas ou três aplicações, ou de heparina de baixo peso molecular (HBPM) dividida em uma ou duas aplicações diárias (enoxaparina 30 mg ou 40 mg). Como a associação de fatores de risco proporciona um risco maior ainda de eventos tromboembólicos nesses pacientes, geralmente associam-se medidas físicas e químicas para ampliar a eficácia da prevenção do TEV 23 . No presente trabalho, observou-se que a totalidade dos 57 pacientes submetidos à cirurgia bariátrica recebeu quimioprofilaxia associada ao método mecânico, revelando que a conduta na instituição está em conformidade com as possibilidades descritas na literatura. 

 Com relação aos pacientes com insuficiência renal, observou-se conformidade no seguimento das recomendações do ACCP, o qual preconiza ajuste posológico para pacientes com *clearance* de creatinina < 30 mL/min, uma vez que existe aumento da exposição ao medicamento e risco de sangramento por acúmulo de fator Xa. A dose plena pode continuar a ser prescrita em casos de insuficiência moderada e leve 24 . 

 Embora sistematicamente anexado ao prontuário no ato da internação, o protocolo institucional foi preenchido para apenas 5% dos pacientes recém-internados, o que deixa a instituição sem conhecimento do perfil de riscos de TEV de seus usuários e menos capaz de tomar medidas mais claras e uniformes no que diz respeito à relação entre estratificação de risco dos pacientes e tromboprofilaxia a ser adotada. Tal situação corrobora achados de outros autores de que a distribuição passiva de protocolos e apenas a divulgação de estratégias tromboprofiláticas apresentam baixa probabilidade de sucesso 5 ^,^ 12 ^,^ 25 . Para esses autores, a implantação de medidas educacionais associadas a outras estratégias de melhoria da qualidade – atuação de comissão multidisciplinar, auditoria e *feedback* em tempo real das recomendações de protocolos – e abordagens tecnológicas de informação como alertas e sistemas informatizados mandatórios de suporte à decisão clínica parecem oferecer opções mais efetivas para promover o uso de boas práticas profiláticas e prevenir danos do TEV aos pacientes. 

 Por tratar-se de estudo transversal realizado apenas no primeiro dia das novas internações, os resultados não podem ser extrapolados quanto à adequação da tromboprofilaxia durante todo o período de internação hospitalar desses pacientes. Porém, apontam para a necessidade premente de programas efetivos que visem a segurança do paciente em relação ao TEV já nas primeiras horas de internação. 

## CONCLUSÕES

 O processo de estratificação identificou que 57% do total de pacientes recém-internados apresentaram indicação para uso da quimioprofilaxia já nas primeiras 24 horas de internação, sendo 42% dos clínicos e 81% dos cirúrgicos. Entretanto, os resultados sobre a conformidade da profilaxia adotada corroboram a literatura e revelam subutilização da profilaxia química para o TEV, tanto no paciente clínico como no cirúrgico. Maior destaque ocorreu em relação aos pacientes clínicos de alto risco e cirúrgicos de moderado risco, uma vez que apenas 54% e 4% desses pacientes, respectivamente, receberam quimioprofilaxia apropriada no período analisado. Há necessidade de aprimoramento da segurança do paciente em relação ao TEV já nas primeiras horas de internação.
